# Identification of Genes Required for Neural-Specific Glycosylation Using Functional Genomics

**DOI:** 10.1371/journal.pgen.1001254

**Published:** 2010-12-23

**Authors:** Miki Yamamoto-Hino, Yoshimi Kanie, Wakae Awano, Kiyoko F. Aoki-Kinoshita, Hiroyuki Yano, Shoko Nishihara, Hideyuki Okano, Ryu Ueda, Osamu Kanie, Satoshi Goto

**Affiliations:** 1 Research Group of Glycobiology and Glycotechnology, Mitsubishi-kagaku Institute of Life Sciences, Tokyo, Japan; 2 Department of Physiology, Keio University, Tokyo, Japan; 3 Mutant Flies Laboratory, Mitsubishi-kagaku Institute of Life Sciences, Tokyo, Japan; 4 Department of Bioinformatics, Faculty of Engineering, Soka University, Tokyo, Japan; 5 Genetic Strains Research Center, National Institute of Genetics, Shizuoka, Japan; Harvard Medical School, Howard Hughes Medical Institute, United States of America

## Abstract

Glycosylation plays crucial regulatory roles in various biological processes such as development, immunity, and neural functions. For example, α1,3-fucosylation, the addition of a fucose moiety abundant in *Drosophila* neural cells, is essential for neural development, function, and behavior. However, it remains largely unknown how neural-specific α1,3-fucosylation is regulated. In the present study, we searched for genes involved in the glycosylation of a neural-specific protein using a *Drosophila* RNAi library. We obtained 109 genes affecting glycosylation that clustered into nine functional groups. Among them, members of the RNA regulation group were enriched by a secondary screen that identified genes specifically regulating α1,3-fucosylation. Further analyses revealed that an RNA–binding protein, second mitotic wave missing (Swm), upregulates expression of the neural-specific glycosyltransferase FucTA and facilitates its mRNA export from the nucleus. This first large-scale genetic screen for glycosylation-related genes has revealed novel regulation of *fucTA* mRNA in neural cells.

## Author Summary

Glycosylation plays crucial regulatory roles in various biological processes such as development, immunity, and neural functions. Accordingly, some glycans are generated in a stage- and tissue-specific manner. To address how such distinct glycosylation is regulated in different tissues, we performed a large-scale screen for genes involved in glycosylation of a neural-specific protein. We identified 109 genes, 95 of which are assigned for the first time as directly or indirectly involved in glycosylation. We further found that neural-specific glycosylation is regulated at the RNA level, which is a novel regulatory mechanism of tissue-specific glycosylation.

## Introduction

Neural cells require correct glycosylation patterns for their development, function, and viability. An example of this is the attachment of an α1,3-fucose moiety to an *N*-glycan core via α1,3-linkage [1], a process which is prevalent in neural cells in *Drosophila* [2]. This α1,3-fucose moiety can be detected with an anti-horse radish peroxidase (HRP) antibody and therefore has also previously been referred to as an HRP epitope. The α1,3-fucose is thought to be essential for neural development, function, and behavior because a *nac* (*neurally altered carbohydrate*) *Drosophila* mutant that lacks this α1,3-fucose moiety exhibits deformation of the eyes [3], the misrouting of wing sensory neurons [4], and abnormal grooming behavior [5]. However, as it remains unclear that the *nac* mutation impairs only α1,3-fucosylation, the necessity of α1,3-fucosylation for neural development and/or function in *Drosophila* has not been conclusively demonstrated.

The enzyme α1,3-fucosyltransferase (FucTA) [6], which is mainly expressed in neural cells, directly catalyzes α1,3-fucosylation. In addition to FucTA, other glycosylation-related proteins such as UDP-GlcNAc: α-3-D-mannoside-β -1,2-N-acetylglucosaminyltransferase I (Mgat1) [7], GDP-mannose 4,6-dehydratase (Gmd) [8], and a GDP-fucose transporter (Gfr) [9], [10] are required for α1,3-fucosylation. Whereas Mgat1 provides a preferred substrate for FucTA by adding *N*-acetylglucosamine to the nonreducing end of an *N*-glycan, Gmd and Gfr are responsible for the synthesis and transport, respectively, of GDP-fucose, another substrate for FucTA. These genes, in contrast to the gene encoding FucTA, are widely expressed in various tissues and also utilized for other glycosylation processes such as *O*-fucosylation of Notch, α1,6-fucosylation of *N*-glycans, and formation of complex type *N*-glycans. Hence, the neural-specific expression of FucTA appears to account for the neural-specific regulation of α1,3-fucosylation. However, the mechanisms regulating FucTA expression have remained largely unknown.

Forward genetic approaches have proven to be powerful methods of elucidating novel mechanisms. For example, the study of *Drosophila* genetics has yielded important contributions to our understanding of the developmental significance of proteoglycans [11], [12] and Fringe-dependent Notch glycosylation [13]. Genetic screens for mutations affecting morphogenesis and growth factor signaling have now identified a number of genes involved in Notch glycosylation and/or proteoglycan formation. Most of these genes are conserved in mammals, suggesting that *Drosophila* is a useful model system for the study of glycosylation in metazoans. However, although previously performed screens of this nature have identified glycosyl enzymes and nucleotide sugar transporters, to date they have not been used to uncover regulators of these molecules.

To elucidate novel regulatory mechanisms underlying neural-specific glycosylation, we performed a genetic screen in *Drosophila* and identified 109 genes required for glycosylation of a retinal neural cell-specific protein. These included 95 genes that are newly implicated in this process and 9 functional groups. Furthermore, 17 genes were identified to be specifically required for α1,3-fucosylation. Among these genes, we further analyzed the function of second mitotic wave missing (Swm), which contains an RNA-binding motif. Here, we show that Swm directly binds to *fucTA* mRNA, upregulates *fucTA* mRNA and protein levels, and facilitates the nuclear export of *fucTA* mRNA in neural cells. These results indicate that Swm is involved in neural-specific glycosylation in addition to the cell cycle, in which its involvement has been previously reported [14]. This report, the first large-scale screen for glycosylation in a multicellular organism, has thus identified a number of new genes directly or indirectly involved in glycosylation and unveiled a novel regulatory mechanism of neural-specific glycosylation.

## Results

### Biological significance of α1,3-fucosylation

**Figure 1:**
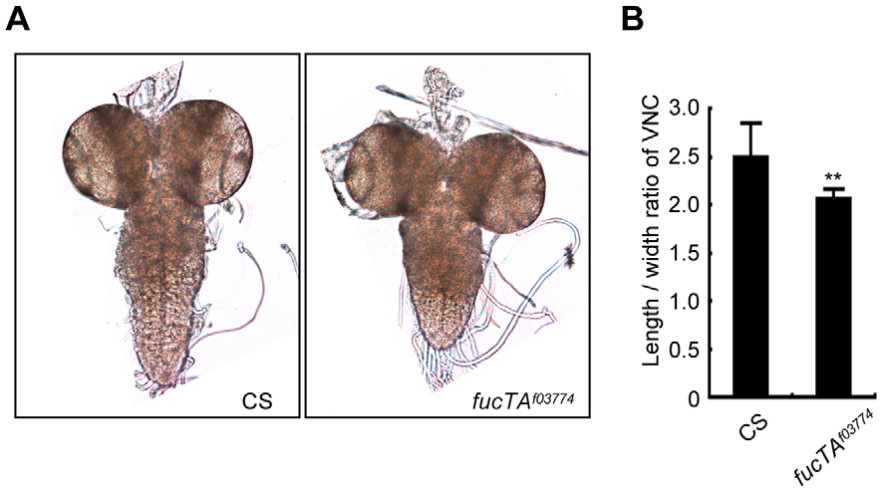
FucTA is required for proper shape of the ventral nerve cord. (A) The wild-type (CS) and *fucTA* mutant (*fucTA^f03774^*) third instar larval CNS. (B) The ratio of longitudinal length to width in *fucTA* mutants was significantly lower that that in wild-type larvae. Results are the mean ± SD (n = 19 CS, n = 10 *fucTA^f03774^* larvae). **p<0.01.

As α1,3-fucosylation has not proven to be essential for neural development and/or function in *Drosophila*, we first aimed to determine the neural function of α1,3-fucosylation. A phenotype of a piggyBac insertional mutant for the *fucTA* gene, *fucTA^f03774^*, was examined. To validate this mutant, its central nervous system (CNS) was stained using anti-HRP antibody. The mutant CNS was negative for anti-HRP staining, suggesting that α1,3-fucosylation was compromised (data not shown). CNS morphology was then compared between wild-type and *fucTA* mutant third instar larvae. The longitudinal length-to-width ratio of the ventral nerve cord (VNC) was significantly lower in *fucTA* mutants than in wild-type larvae (Figure 1A and 1B), indicating that α1,3-fucosylation plays an important role in neural development. However, the staining patterns of antibodies such as 22C10, BP102, anti-FasI, anti-FasII, and anti-FasIII were not noticeably different in the *fucTA* mutant (data not shown).

### Screening strategy

**Figure 2:**
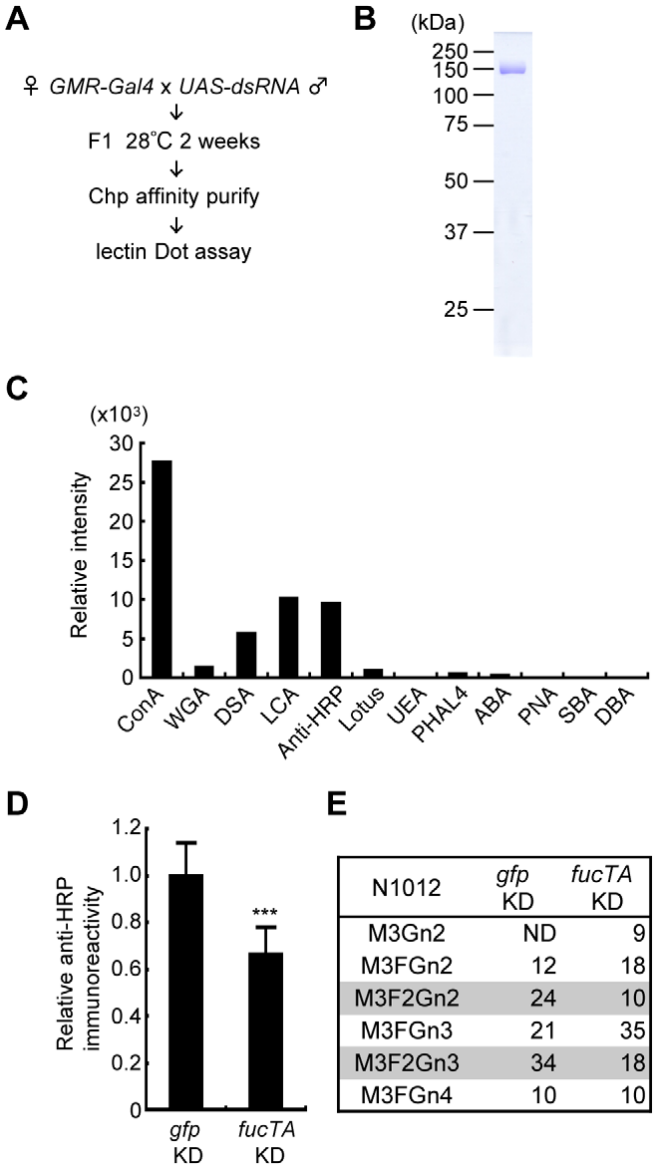
Screening strategy and validation of the primary screen for genes whose knockdown impairs glycosylation. (A) Outline of screen design. (B) Coomassie Brilliant Blue (CBB) staining of Chp affinity-purified with anti-Chp antibody and separated by SDS-PAGE. Chp could be purified as a single band (∼150 kDa) by a single step of affinity purification. (C) Binding abilities of glycan probes to purified Chp. The signal intensities normalized to the amount of Chp are represented. (D) Anti-HRP antibody reactivity normalized to Chp levels was decreased in *fucTA* knockdown flies (*fucTA* KD) compared to that in control *gfp* knockdown flies (*gfp* KD). Results are the mean ± SD of 4 experiments. ***p<0.005. (E) Glycan structures attached to N1012 of Chp revealed by mass spectrometry analysis. Percentages of each structure in the control (left, *gfp* KD) and *fucTA* knockdown conditions (right, *fucTA* KD) are listed. The glycans bearing α1,3-fucose are highlighted (gray). Schematic structures are represented in Figure S3. M, mannose; F, fucose; Gn, *N*-acetylglucosamine. ND, not detected.

To reveal novel regulatory mechanisms underlying neural-specific glycosylation, a genetic screen was performed in adult *Drosophila* using the RNAi-mediated tissue-specific gene knockdown method. The procedure is summarized in Figure 2A. We chose eye-specific knockdown because eyes bear the neural-specific glycan α1,3-fucose. Additionally, the ablation of genes essential for development and/or cell viability in eyes might not cause knockdown fly lethality, whereas their ablation in the central and/or peripheral nervous systems would be lethal. DsRNAs targeting different genes were expressed in *Drosophila* eyes by crossing the eye-specific *Gal4* driver, *GMR-Gal4*, with the corresponding strains. These strains harbored inverted repeats of a portion of the corresponding cDNA downstream of the yeast upstream activating sequence (UAS), which is activated by the Gal4 protein (http://www.shigen.nig.ac.jp/fly/nigfly/about/aboutRnai.jsp).

In our screen, the glycosylation of a model eye-specific glycoprotein was examined, as this allowed us to accurately measure the number of glycans added. We chose Chaoptin (Chp) [15] as our model glycoprotein based on the following advantages: (i) Chp is modified by neural-specific α1,3-fucosylation; (ii) detailed information is available on both the structures of the *N*-glycans and their attachment sites on Chp (described below in detail) [16]; and (iii) simple protein affinity purification is possible using an anti-Chp antibody (24B10). As shown in Figure 2B, Chp protein could be purified as a single band detected by Coomassie Brilliant Blue (CBB) staining.

Chp is a *Drosophila* glycoprotein essential for rhabdomere formation in adult photoreceptor cells, which is mediated through cell adhesion activity [15], [17]. *N*-glycosylation of Chp was recently shown to play a pivotal role in its stability, transport to the plasma membrane, and cell adhesion activity [18]. Chp *N*-glycan structures are classified as high-mannose type, pauci-mannose type, or complex type [16]. Glycan structures added to Chp are summarized in Figure S1. Interestingly, α1,3-fucose is added only at N1012, suggesting that Chp has a single difucosylated glycan at N1012. To detect these glycan structures, the binding of glycan probes such as lectins and anti-HRP antibody was examined. Immunopurified Chp was subjected to lectin blot or immunoblot analysis using ABA, ConA, DBA, DSA, LCA, Lotus, PHA-L4, PNA, SBA, UEA, and WGA lectins and anti-HRP antibody. The specificity of these lectins and anti-HRP antibody is summarized in Table S1. The binding abilities of these probes were quantified relative to the amount of Chp protein (Figure 2C). These analyses suggested that the WGA, ConA, LCA, and DSA lectins and anti-HRP antibody would be useful as glycan probes in our assay system. As represented in Figure S1, WGA and ConA detect *N*-acetylglucosamine (GlcNAc) and mannose moieties of *N*-glycan core regions, respectively. LCA preferentially binds to *N*-glycans with α1,6-fucose and even weakly recognizes the core regions of *N*-glycans. Anti-HRP antibody binds to the α1,3-fucose moieties attached to the core [1], [19]. DSA recognizes GlcNAc at the non-reducing end of *N*-glycans.

Chp protein purified from knockdown flies was subjected to lectin and immuno-dot blot analyses using the same glycan probes described above and an anti-Chp antibody. An example of the dot blot analysis is shown in Figure S2. The glycan moieties detected by these probes were quantified relative to Chp protein, and the *z*-scores for each knockdown experiment were thus calculated. For verification of this procedure, a dsRNA that targets FucTA was expressed. The resulting knockdown of *fucTA* decreased the affinity of Chp to the anti-HRP antibody (66.2±11.8% of the control, Figure 2D). Given the results of our previous mass spectrometry study showing that α1,3-fucose is added to the Chp N1012 site [16], Chp was purified from *fucTA* knockdown eyes, and mass spectrometry analysis was performed. The glycan structures attached to N1012 are summarized in Figure S3. The number of glycans with two fucoses present at N1012 (M3F2Gn2 and M3F2Gn3) selectively decreased in *fucTA* knockdown eyes (Figure 2E). This finding is consistent with those of previous studies reporting that most single- or double-fucosylated *N*-glycans bear α1,6-fucose alone, or both α1,6- and α1,3-fucose, respectively, in embryos [20] and adults [6].

### Identification of glycosylation-related genes

A large-scale screen was performed in the current study using an RNAi library that was previously constructed in National Institute of Genetics, Japan (NIG, http://www.shigen.nig.ac.jp/fly/nigfly/about/aboutRnai.jsp). With this library, the expression of 6923 *Drosophila* genes can be suppressed. From the primary screen followed by tests for reproducibility, 171 genes were identified as candidate genes, knockdown of which compromised glycosylation (Table S2). Since these candidate genes likely included false-positives due to the off-target effects of RNAi [21], the results were validated by repeating the knockdown experiments using secondary sets of dsRNAs. During the construction of these secondary sets of dsRNAs, we ensured that the target regions did not overlap.

Of the 171 primary candidate genes, only 80 could be tested because, in most cases, the induction of the secondary sets of dsRNA resulted in lethal or severe eye malformation or cDNAs were too short to permit the design of secondary target regions. Of the 80 genes tested successfully, 57 were verified to be involved in glycosylation. Sources of the RNAi strains used for the verification of the 57 genes are listed in Table S2 (see “Line ID or transformant ID used for validation”). When the validation results were compared with the calculated off-target probability scores (OTPS) provided by dsCheck software [22], 95.6% of the validated genes had scores of less than 3, whereas the scores of 60.7% of the genes yet to be validated were greater than 2. Thus, the genes with OTPS less than 3 were rescreened by testing whether knockdown of their suspected off-target genes led to glycosylation defects. When knockdown of the suspected off-target genes did not induce any glycosylation defects, the corresponding genes were classified as glycosylation-related genes (rank 2) along with the genes that were validated by secondary dsRNAs (rank 1) (Table S4).

To successfully identify additional glycosylation genes, we searched for genes that interacted with our newly identified genes in the yeast two-hybrid database BIOGRID (http://www.thebiogrid.org/) and in the genetic interaction data listed in FlyBase (http://flybase.bio.indiana.edu/). The RNAi fly strains harboring candidate genes that did not already exist in our library were obtained from the Vienna *Drosophila* RNAi Library Center (VDRC) [23] (Table S3). Among the 186 genes tested, 10 genes showed glycosylation defects. Further validation experiments identified 1 rank-1 and 5 rank-2 glycosylation-related genes among them. In total, 109 genes were eventually isolated and identified as glycosylation-related genes (Table S4). Significantly, although 14 of the 109 identified genes have already been shown to be involved in glycosylation, the remaining 95 genes were newly assigned to this category by our analysis.

### Clustering of glycosylation genes

**Figure 3:**
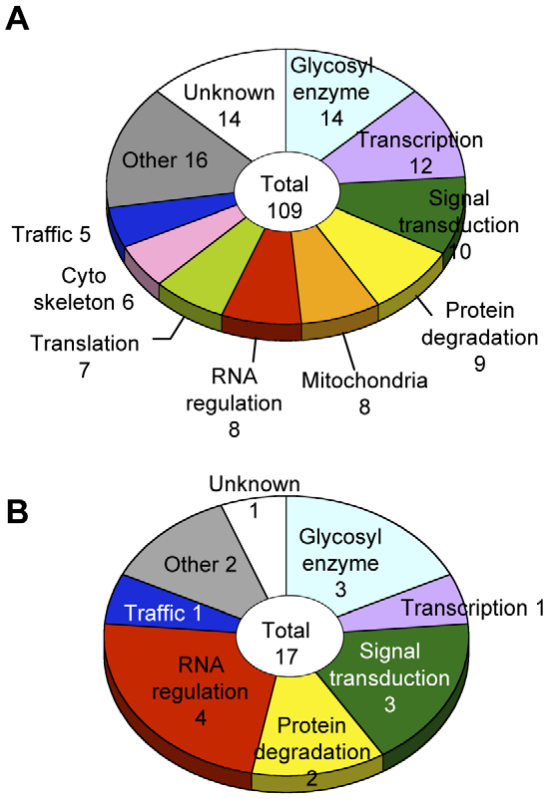
Screening results. (A) Functional classification of the 109 glycosylation-related genes. (B) Functional classification of the 17 α1,3-fucosylation-specific glycosylation-related genes. The number of genes in each category is listed.

Glycosylation-related genes were classified based on their domain structures and presumptive functions using the InterPro (http://www.ebi.ac.uk/interpro/), FlyBase (http://flybase.org/), and Panther (http://www.pantherdb.org/) databases. The gene functional groups obtained included glycosylation reactions, transcription, RNA regulation, translation, intracellular trafficking, cytoskeletal regulation, signal transduction, protein degradation, mitochondrial function, and other functions (Figure 3A, Table S4).

### Genes regulating neural-specific glycosylation

To identify genes specifically involved in neural-specific glycosylation, we selected genes whose knockdown induced only abnormal anti-HRP binding activity from among the 109 genes listed in Table S4. From this analysis, 17 genes showing α1,3-fucosylation-specific defects were identified belonging to seven functional groups (Figure 3B). In the RNA regulation group, *swm* (CG10084) [14], *Pabp2* (CG2163) [24], and *Hel25E* (CG7269) are suggested to interact with each other in the *Drosophila* interaction database STRING (http://string.embl.de/) and literature [25].

### Swm and Pabp2 are required for α1,3-fucosylation

**Figure 4:**
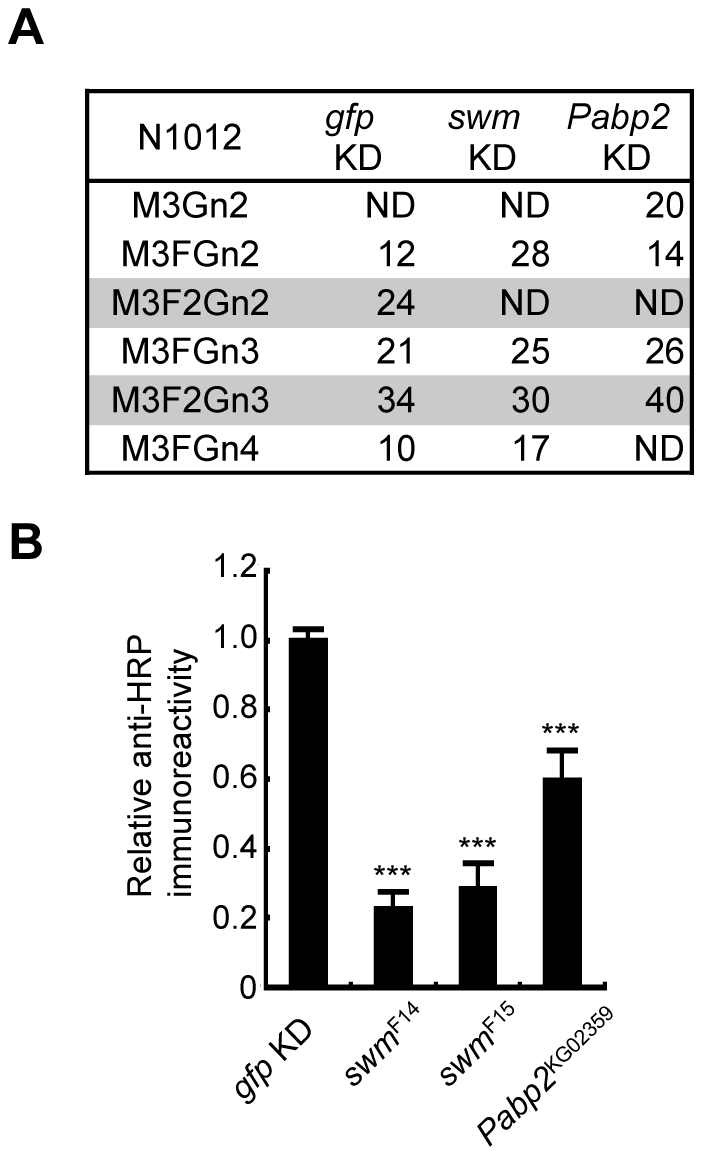
Swm and Pabp2 are required for neural-specific α1,3-fucosylation. (A) Glycan structures attached to N1012 of Chp as revealed by mass spectrometry analysis. The percentages of each structure in the control (left, *gfp* KD), *swm* (middle, *swm* KD), or *Pabp2* (right, *Pabp2* KD) knockdown condition are listed. The glycans bearing α1,3-fucose are highlighted (gray). ND, not detected. (B) α1,3-fucosylation in *swm* and *Pabp2* mutants. Anti-HRP antibody reactivity normalized to Chp levels was markedly decreased in *swm^F14^*, *swm^F15^*, and *Pabp2^KG02359^* mutants. Results are the mean ± SD of 3 experiments. ***p<0.005.

Since Swm and Pabp2 have been reported to physically interact with each other in BIOGRID, the roles of these genes were further analyzed in the context of glycosylation. To analyze the glycosylation defects induced by the knockdown of these genes in further detail, Chp glycans in the *swm* and *Pabp2* knockdown lines were subjected to mass spectrometric analysis. The ratio of the α1,3-fucosylated form (M3F2Gn2) at N1012 was markedly decreased in both knockdown lines, whereas the other form (M3F2Gn3) was only slightly affected by *swm* or *Pabp2* knockdown (Figure 4A). These results suggest that Swm and Pabp2 are required for α1,3-fucosylation and that the M3F2Gn2 form might be more sensitive than the M3F2Gn3 form to decreases in Swm and Pabp2.

Since *swm* and *Pabp2* mutants have been previously isolated [26] (Flybase), glycosylation defects were also examined in the amorphic and hypomorphic alleles of *swm*, *swm^F14^* and *swm^F15^*, respectively, and the hypomorphic allele of *Pabp2*, *Pabp2^KG02359^*. All cells homozygous for *swm* mutations were generated in whole *Drosophila* eyes using a modified FLP/FRT system [27]. Chp was isolated from these mutant eyes and detected by immunoblotting with anti-Chp and anti-HRP antibodies to quantify the amount of α1,3-fucose added to Chp. The α1,3-fucose added in *swm^F14^*, *swm^F15^*, and *Pabp2^KG02359^* cells decreased to 22.7% ± 4.8%, 28.3% ± 7.3%, and 59.5% ± 8.8% of wild-type levels, respectively (Figure 4B). These results are further evidence from knockdown experiments that Swm and Pabp2 are somehow required for α1,3-fucosylation.

**Figure 5:**
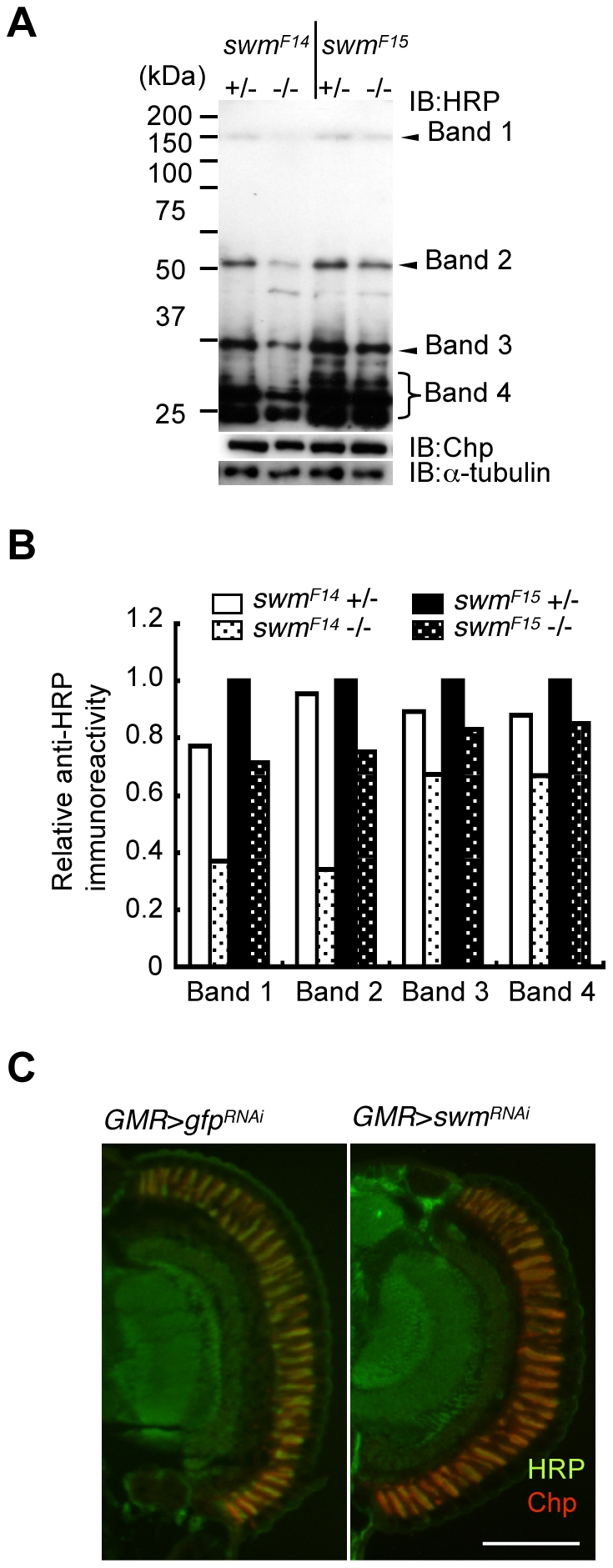
Immunoblot analysis. (A) Immunoblot analysis of whole-eye extracts from *swm^F14^* and *swm^F15^* mutants (*swm^F14^* and *swm^F15^*). The anti-HRP antibody detected several bands, including Chp (Band 1). Anti-HRP antibody reactivity was decreased in *swm* mutant homozygotes (−/−) compared with heterozygotes (+/−). Loading controls with anti-Chp and α-tubulin antibodies are shown. (B) Quantified immunoblot band intensity. HRP-positive band intensities were normalized to the intensities of corresponding anti-α-tubulin signals. The values normalized to those in *swm^F15^* heterozygotes (+/−) are represented. (C) Anti-HRP signal (green) relative to anti-Chp signal (red) in *swm* knockdown mutants (*GMR* > *swm^RNAi^*) was obviously reduced compared to that in control knockdown mutants (*GMR* > *gfp^RNAi^*). Scale bar: 100 µm.

Although Pabp2 is known to participate in polyA addition, the function of Swm is largely unknown. Thus, we focused on the role of Swm in glycosylation. Modification of α1,3-fucose is widely utilized for *Drosophila* neural proteins. Thus, we first examined whether *swm* depletion affects not only Chp but also a wide range of other proteins. Homogenates of adult eyes heterozygous or homozygous for *swm^F14^* and *swm^F15^* were analyzed by immunoblot analysis with an anti-HRP antibody (Figure 5A). Several positive bands were detected, including a high-molecular-weight band corresponding to Chp (∼150 kDa). Quantitative comparison between heterozygotes and homozygotes of the same *swm* alleles revealed that anti-HRP antibody affinity was reduced in most bands in the homozygotes, with the exception of an approximately 30-kDa product (Figure 5A and 5B). The decrease was much larger in *swm^F14^* than in *swm^F15^* mutants, consistent with the severity of these mutations. Taken together, our data indicate that Swm is required for the α1,3-fucosylation of most proteins that are usually modified by α1,3-fucose.

The observed requirement of Swm for most α1,3-fucosylation was also supported by the immunostaining of eyes with anti-HRP antibody. Adult photoreceptor cells from *swm* knockdown and control flies were simultaneously stained with anti-HRP and anti-Chp antibodies (Figure 5C). Control photoreceptor cells appeared yellow in color because the intensities of green signal (anti-HRP) and red signal (anti-Chp) were nearly equal in this condition. In contrast, *swm* knockdown cells appeared relatively red in color, suggesting that the intensity of green signal was reduced compared to the red signal. These data clearly indicate that the signal strength of anti-HRP relative to anti-Chp was reduced in the knockdown photoreceptor cells compared to control cells.

**Figure 6:**
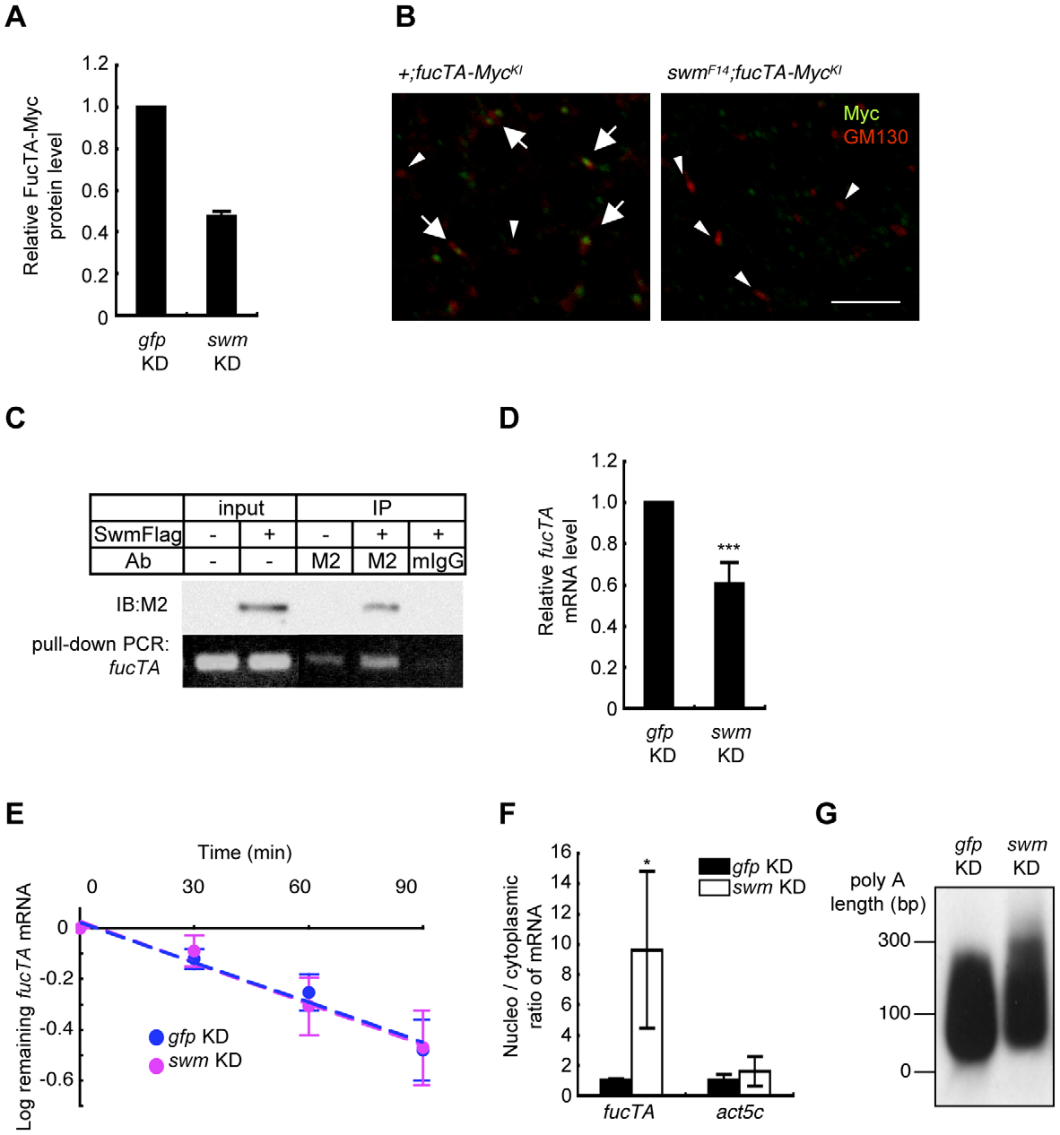
Swm regulation of expression and nuclear export of *fucTA* mRNA. (A) Quantification of FucTA-Myc in control (*gfp* KD) and *swm* (*swm* KD) knockdown eyes. In this figure, all results shown in graphs are the mean ± SD, *n* = 2. (B) Expression of FucTA-Myc in the Golgi complex was reduced in *swm^F14^* mutants. FucTA-Myc (green) was well colocalized to the Golgi detected by anti-GM130 (red) in control eyes (arrows, +;*fucTA-Myc^KI^*). In contrast, FucTA-Myc was hardly colocalized to the Golgi (arrowheads) in *swm* mutants (*swm^F14^*; *fucTA-Myc^KI^*). Scale bar: 10 µm. (C) *In vivo* binding of Swm and endogenous *fucTA* mRNA. Endogenous *fucTA* mRNA was immunoprecipitated with an anti-Flag antibody (M2) from BG2-c6 cells expressing Swm-Flag and detected by RT-PCR (bottom). Control experiments were performed without Swm-Flag expression (−) or by immunoprecipitation with a mouse IgG antibody (mIgG). (D) Decrease of *fucTA* mRNA in *swm* knockdown BG2-c6 cells. Levels of *fucTA* RNA in *swm* knockdown cells was normalized to that in control *gfp* knockdown cells, *n* = 3. ***p<0.005. (E) Decay of *fucTA* mRNA in *swm* knockdown BG2-c6 cells. Levels of *fucTA* mRNA at each time point after actinomycin D addition were normalized to those at time 0, *n* = 3. (F) Ratios of *fucTA* and *actin* (act5c) mRNA levels in nuclear vs. cytoplasmic fractions in *swm* knockdown BG2-c6 cells were normalized to those in control *gfp* knockdown cells. *n* = 4. (G) PolyA length of *fucTA* mRNA in *swm* knockdown BG2-c6 cells. PolyA of *fucTA* was amplified by PCR and detected by Southern blotting with a probe corresponding to *fucTA*.

Since α1,3-fucosylation is directly catalyzed by FucTA, the Swm-dependence of FucTA protein expression was examined. Because no anti-FucTA antibody was available, a knock-in fly was generated in which the *fucTA* locus was replaced by a Myc-tagged *fucTA* gene (Figure S4A and S4B). When *swm* dsRNA was expressed by *GMR-Gal4*, FucTA-Myc protein levels were reduced to 47.5±2.4% of control values as revealed by immunoblot analysis (*n* = 2) (Figure 6A and Figure S4C). The reduction of FucTA-Myc levels in *swm* mutant tissue was also examined. We costained the CNS of first instar larvae with anti-Myc and anti-GM130, a Golgi marker. The number of Golgi harboring FucTA-Myc protein was markedly decreased in *swm^F14^* mutants (2.0% of total Golgi) compared to wild-type larvae (43.1% of total Golgi, Figure 6B). The different ratios of FucTA-Myc reduction observed in the biochemical and immunostaining experiments may be due to different levels of Swm depletion in knockdown and mutant flies.

### Functional analyses of Swm

Although Swm has an RNA recognition motif (RNP-1) that is present in a wide variety of other RNA binding proteins [26], it remained undetermined whether Swm directly bound to mRNA. Binding of Swm to *fucTA* mRNA was thus examined by expressing Flag-tagged Swm in the *Drosophila* neural cell line BG2-c6 [28] and by performing immunoprecipitation with an anti-Flag antibody. The quantity of endogenous *fucTA* mRNA that coprecipitated with Swm-Flag was higher in Swm-Flag–expressing cells than in controls in which Swm-Flag was not expressed or immunoprecipitation was performed using a control IgG (Figure 6C). These results strongly suggest that Swm binds to *fucTA* mRNA *in vivo*.

The amount of *fucTA* mRNA under the control of Swm was next examined. dsRNA targeting *swm* was introduced into BG2-c6 cells, and the amount of endogenous *fucTA* mRNA was measured. In addition to a decrease in *swm* mRNA, to 27.3 ± 1.9% of the control level (*n* = 3), the amount of *fucTA* mRNA also decreased to 60.6 ± 10.5% of the control level (Figure 6D). Furthermore, the stability of *fucTA* mRNA was examined in *swm* knockdown cells. The amount of *fucTA* mRNA was measured at 30, 60, and 90 min after total transcription was inhibited by addition of actinomycin D. Decay rates of *fucTA* were indistinguishable between *swm* knockdown and control cells (swm, *t*_1/2_ = 97.72; control, *t*_1/2_ = 100.48, Figure 6E). These data suggest that Swm is involved in the expression of *fucTA* mRNA but not in its stability.

Swm was recently reported to incorporate into the complex that regulates nuclear export of mRNAs [29]. The necessity of Swm for the nuclear export of *fucTA* mRNA was therefore examined in BG2-c6 cells. Following separation of cell lysates into nuclear and cytoplasmic fractions, *fucTA* mRNA levels were measured in each fraction of *swm* knockdown and control cells. The amount of *fucTA* mRNA in the nucleus increased by 9.6-fold in *swm* knockdown cells compared to control *gfp* knockdown cells (Figure 6F). The nuclear export of *actin* (*act5c*) mRNA was also examined in *swm* knockdown cells. The amount of *actin* mRNA in the nucleus increased by 1.7-fold in *swm* knockdown cells compared to control cells. However, the fold change in nuclear accumulation of *actin* mRNA was significantly lower than that of *fucTA* mRNA in *swm* knockdown cells (Figure 6F). These results suggest that relatively more Swm is required for nuclear export of *fucTA* mRNA than for nuclear export of *actin* mRNA.

Since nuclear export defects have been reported to result in an abnormally longer polyA length of mRNAs [29], the polyA length of *fucTA* mRNA was examined in *swm* knockdown cells. The polyA length of *fucTA* mRNA was clearly longer in *swm* knockdown cells than in control cells (Figure 6G). Collectively, these results indicate that Swm positively regulates α1,3-fucosylation by promoting nuclear export of *fucTA* mRNA in neural cells.

## Discussion

The current literature on glycosylation regulatory mechanisms is remarkably limited, and our current study is in fact the first report of a large-scale screen for glycosylation genes using a multicellular organism. We identified 109 genes in our screen, including 95 genes that have not been previously associated with glycosylation. *In silico* analyses using the available *Drosophila* databases suggested that these gene products participate in a variety of cellular processes (Figure 3), among which RNA regulation is, for the first time, shown to play an important role in the context of glycosylation. Moreover, the candidate genes belonging to the RNA regulation group were enriched by rescreening for α1,3-fucosylation-specific genes.

### Biological significance of α1,3-fucosylation

In the current study, the *fucTA* mutant showed an imbalanced VNC. The VNC longitudinal length-to-width ratio was significantly shorter in *fucTA* mutants compared to wild-type larvae. To our knowledge, this is the first report of such shortened VNC in the loss-of-function mutant, although elongated VNC has already been reported in mutants for *integrin* [*30*], *collagen IV* [*31*], *DHR3* [*32*], *worniu* [*33*] and *C1GalTA* [*34*]. Interestingly, *C1GalTA* encodes a core 1 galactosyltransferase that adds galactose to *O*-GalNAc attached to Ser or Thr protein residues. Taken together with our results, glycosylation patterns might affect the VNC shape. Although how α1,3-fucose affects VNC formation remains to be revealed, we think that two possible defects may result in a shortened VNC in *fucTA* mutants. (1) The aberrantly elongated VNC in the *C1GalTA* mutant is proposed to result from deglycosylation of laminin, a component of the extracellular matrix. In addition, overexpression of FucTA has recently been reported to enhance cell migration in the embryonic nervous system [35]. By analogy with the case of C1GalTA, cell migration in the larval VNC may be impaired in *fucTA* mutants because of aberrant interactions between migrating cells and extracellular matrix. (2) Another possibility is that neural activity may be impaired in *fucTA* mutants because neural activity has been reported to be necessary for VNC condensation in embryos [31]. This possible defect is consistent with the finding that one of the major α1,3-fucosylated proteins is a β-subunit of Na^+^/K^+^ ATPase essential for the maintenance of neural cell membrane potentials [36]. It will be interesting to examine whether neural activity is controlled by glycosylation in the future.

### Insight into the genes identified in this screen

Three types of genes were identified in this screen: (1) genes that specifically regulate glycosylation such as glycosyltransferases, (2) genes with multiple functions including regulation of expression, activity and/or localization of glycosyltransferases, and (3) genes with primary functions other than glycosylation that secondarily affect glycosylation. For example, knockdown of cytoskeletal regulation and mitochondrial function genes may secondarily affect glycosylation as a result of damage to cell structures and viability.

For several reasons, *swm* might be the second type of gene. Although *swm* has been previously reported to show involvement in the cell cycle [14], no glycosylation defects were observed with the knockdown of other known cell cycle regulators such as *cdc2* (CG5363) or *cdk4* (CG5072) (Table S2 and S3). In addition, no cell cycle defects could be detected in the *fucTA* mutant. These data suggest that cell cycle and glycosylation are regulated independently. Therefore, *swm* would play at least two independent roles in cell cycle and glycosylation.

Traffic genes identified in this screen may be another example of the second type of genes. The conserved oligomeric Golgi (COG) complex has been reported to participate in the Golgi localization of glycosyltransferases and membrane traffic [37], but no involvement of the COG complex in glycosylation has been reported in *Drosophila*. Our screen identified *Cog3* as a gene whose knockdown impaired α1,3-fucosylation. Thus, localization of FucTA protein was examined in *cog3* knockdown photoreceptor cells. Expressed FucTA was hardly localized to the Golgi complex in knockdown photoreceptor cells (data not shown). Moreover, Golgi localization of FucTA was also preliminarily found compromised by knockdown of *vps2* and *vps20*, other traffic genes (data not shown). These data raise the possibility that some traffic genes may be involved in glycosylation by localizing glycosyltransferases to appropriate Golgi and/or endoplasmic reticulum sites.

### Preferential requirement of Swm in glycoforms

Our detailed analysis of glycan structures using mass spectrometry revealed that one (M3F2Gn2) of the two forms bearing an α1,3-fucose (M3F2Gn3 and M3F2Gn2) is more sensitive to the decrease of *swm* and *Pabp2* than the other (M3F2Gn3). Two reasons may account for this difference, which are not mutually exclusive. (1) FucTA productivity differs between these two glycoforms. The structural difference between the glycoforms consists of a GlcNAc moiety attached to the nonreducing end of *N*-glycans. Given that this GlcNAc moiety has been shown to increase the reactivity of FucTA to the core of *N*-glycans [6], we assume that M3FGn3 is preferentially modified by FucTA to form M3F2Gn3. Therefore, under the condition of reduction, not complete loss, of FucTA by *swm* and *Pabp2* knockdown, M3F2Gn2 might be preferentially lost. (2) The other possibility is that Swm or Pabp2 regulate Mgat1 or Fused lobes (Fdl) [38], the enzymes that add and remove the GlcNAc moiety, respectively, from the nonreducing end. In fact, the amount of mRNA encoding *Mgat1*, but not *fdl*, was slightly but significantly increased in *swm* knockdown cells (Figure S5). This finding is consistent with the selective decrease of M3F2Gn2 induced by *swm* knockdown. However, it remains undetermined whether the preferential decrease of glycoforms is caused by an increase in Mgat1, the preferential productivity of FucTA, or both.

### Regulation of *fucTA* mRNA by Swm

Expression of mRNA is generally regulated at transcription, splicing, 5′- or 3′-end processing, and nuclear export, and by the stability of the mRNA. The stability of *fucTA* mRNA was not affected by *swm* knockdown (Figure 6E). Given that 5′-end processing is essential for mRNA stability, the 5′-end processing of *fucTA* mRNA is not regulated by Swm. In addition, splicing of *fucTA* mRNA does not appear to depend on Swm activity since the misspliced form of *fucTA* mRNA in the *swm* knockdown cells could not be detected when we amplified *fucTA* mRNA using PCR primers that targeted different exons (data not shown). Furthermore, both transcription and nuclear export of mRNAs are regulated by the THO/TREX complex [39] and Sus1 [40]; thus, Swm may be a factor participating in both processes, with its depletion resulting in reduced expression and defective nuclear export of *fucTA* mRNA. We assume that abnormal extension of polyA in *swm* knockdown cells would be a secondary effect of nuclear export defects, as observed in mutants of genes required for nuclear export [29]. Moreover, Pabp2, Hel25E/UAP56 [41], and Nup358 [42], which participate in the nuclear export of mRNA, were also identified in our screen (Table S4), suggesting that nuclear export might be essential for glycosylation regulation.

Nuclear accumulation of polyA(+) RNA in *swm* knockdown S2 cells was confirmed in our *in situ* hybridization experiment, as previously reported [43]. However, in neural BG2-c6 cells, weak polyA(+) RNA signals in nuclei were comparable between control *gfp* knockdown and *swm* knockdown cells (data not shown), suggesting that only a small portion of mRNA might be exported by Swm. In addition, the ratio of nuclear to cytoplasmic *fucTA* mRNA was relatively higher than that of *actin* mRNA in *swm* knockdown BG2-c6 cells (Figure 6F). Collectively, Swm may have preferred mRNAs for nuclear export. Moreover, the export efficiency and/or target RNA preference of Swm may differ between S2 and BG2-c6 cells.

### Mammalian homologs of genes identified in this screen

Most of the genes identified in our present study have mammalian homologs (Table S5). Among these, Cog3 has been reported to be involved in glycosylation in organisms ranging from yeast to humans [37]. Interestingly, mutations of some COG components are causative for human diseases including congenital disorders of glycosylation (CDG) [44]. Further investigation of the genes identified here will likely provide additional insights into novel glycosylation regulatory mechanisms conserved in organisms ranging from *Drosophila* to humans and, possibly, into diseases involving glycosylation pathways.

## Materials and Methods

### Fly stocks and clone analysis

The following fly strains were used: *Canton-S* as the wild-type strain, *GMR-Gal4* (Bloomington *Drosophila* Stock Center (BDSC)), *fucTA* mutant *Pbac{WH}FucTAf03774* (BDSC), *swm* mutants *swm^F14^* and *swm^F15^*(BDSC), and *Pabp2* mutant *P{SUPor-P}Pabp2{KG02359}* (BDSC). RNAi lines were mainly supplied by the National Institute of Genetics (Japan), with the remainder purchased from the Vienna *Drosophila* RNAi Center (VDRC). The transgenic flies harboring *UAS-dicer2* were also purchased from VDRC.

The *swm* and *Pabp2* mutant mosaic clones were generated using the FLP-FRT system [27]. Adult heads from a cross of w[*]; P{w[+t*] ry[+t*] = white-un1}30C swm[F14] P{ry[+t7.2] = neoFRT}40A/CyO, P{Wee-P.ph0}2 or w[*]; P{w[+t*] ry[+t*] = white-un1}30C swm[F15] P{ry[+t7.2] = neoFRT}40A/CyO, P{Wee-P.ph0}2 with y[1]w[*]; P{w[+mC] = GMR-hid}G1 P{ry[+t7.2] = neoFRT}40A, l(2)CL-L[1]/CyO; P{w[+m*] = GAL4-ey.H}SS5, P{w[+mC] = UAS-FLP1.D}JD2 (BDSC), or y[1]; P{y[+mDint2] w[BR.E.BR] = SUPor-P}Pabp2[KG02359] P{neoFRT}42D/SM1 with y[1], w[*]; P{ry[+t7.2] = neoFRT}42D P{y[+t7.7] ry[+t7.2] = Car20y}44B, P{w[+mC] = GMR-hid}SS2, l(2)CL-R[1]/CyO; and P{w[+m*] = GAL4-ey.H}SS5, P{w[+mC] = UAS-FLP1.D}JD2 (BDSC) were dissected and used for glycosylation analyses as described below.

To investigate the localization of FucTA in RNAi-mutant flies, inducible Myc-tagged FucTA-expressing flies (*UAS-fucTA-Myc*) were generated. *fucTA* cDNA was a gift from S. Nishihara. A DNA fragment encoding the 3*Myc* sequence was ligated to the 3′end of the coding region of *fucTA* and inserted into the pUAST vector. The vector was injected into *w^1118^* embryos for the production of transgenic flies.

To generate *fucTA-Myc* knock-in flies (*fucTA-Myc^KI^*), the procedure was adopted from the ends-out knock in method [45]. Details are shown in Figure S4.

### Antibodies and lectins

Commercial sources provided biotin-conjugated Lotus; HRP-conjugated ConA, WGA, DSA, LCA, UEA, PHAL4, PNA, SBA, and DBA lectins (Seikagaku Kogyo); and HRP-conjugated ABA (Sigma). Anti-HRP antibody was obtained from Jackson Immunoresearch. In addition, the antibodies used in this study are as follows: streptavidin-POD (Roche); anti-Myc A14 and anti-Myc 9E10 (Santa Cruz); anti-Flag M2 (Sigma); HRP-conjugated anti-mouse IgG, HRP-conjugated anti-rabbit IgG, and normal mouse IgG (Jackson Immunoresearch); Alexa 488-conjugated anti-mouse IgG, Alexa 488-conjugated anti-rabbit IgG, and TOPRO3 (Invitrogen); and Cy3-conjugated anti-mouse IgG and Cy3-conjugated anti-rabbit IgG (Kirkegaard & Perry Laboratories, Inc.). Anti-Chp 24B10, anti-α-tubulin, 22C10, BP102, anti-FasI, anti-FasII, and anti-FasIII were obtained from the Developmental Studies Hybridoma Bank. Anti-Chp antibody N8A [18] and anti-GM130 [46] were generated as described previously.

### Primers

The primer sets used for the generation of transgenic flies are listed in Table S6. The primer sets used for analysis in cultured cells are listed in Table S7.

### Quantification of ventral nerve cord shape

The central nervous systems were manually isolated from CS and *fucTA^f03774^* third instar larvae and fixed in 4% paraformaldehyde/PBS at 4°C overnight. After several washes with PBS, brain hemispheres were manually removed and placed flatly onto slide glasses. The images were captured using an AxioCam-equipped Zeiss AxioImager M1, and the length and width of VNCs were measured using AxioVision Rel. 4.7 software.

### First screen

The first screen was carried out using a collection of transgenic fly lines carrying inducible dsRNA constructs. To drive expression of dsRNA for each gene in the eye, males from each RNAi fly line were crossed with *GMR-Gal4* virgin females. Mating to inducible RNAi males and egg laying were carried out at 25°C for two days, and the resulting embryos were then incubated at 28°C. After 2 weeks, the hatched knockdown flies were collected and cultured for an additional 14 days at 28°C to enhance the effects of dsRNAs. More than 100 heads per line were isolated and homogenized in lysis buffer consisting of 10 mM sodium phosphate (pH 7.0), 100 mM NaCl, 0.5% Triton X-100, and complete protease inhibitor (Roche). The lysate was incubated with anti-Chp (24B10) covalently coupled with protein G-Sepharose (GE Healthcare) for 2 h. The Chp-bound Sepharose was subsequently washed with lysis buffer, and Chp was eluted with 50 mM triethylamine-acetic acid (pH 3.5).

Purified Chp was spotted onto Hybond ECL nitrocellulose membranes (GE Healthcare) using a Bio Dot slot format (Bio-Rad). After the membrane was blocked with 2% bovine serum albumin, immuno- or lectin blot analysis was performed with anti-Chp (N8A), anti-HRP, or HRP-conjugated ConA, LCA, DSA, and WGA. HRP-conjugated anti-mouse or HRP-conjugated anti-rabbit IgG was used as the secondary antibody. Detection was performed using Supersignal West Pico Chemiluminescent Substrate (Thermo), and quantification performed using a LAS-1000 Luminescent Image Analysis System (FujiFilm). An example of the dot blot is shown in Figure S2. Lectin affinity values per Chp values were converted to log values for normalization. On the basis of standard deviations from the values of *GMR>GFP^RNAi^* (as a control), genes with a *z*-score >3 were considered primary candidates. In the primary screen, we performed a single assay round. For the primary candidates, the lectin analysis was repeated (*n* = 3). Two-sided Student's *t*-tests were applied to the reproducibility test data between lectin values of knockdown and control flies. Genes with *p*<0.005 were considered to be reproducible findings.

### Validation

Collection of the second transgenic fly stock lines was designed in such a way that there was no overlap between the primary construct in the original library and the new dsRNA sequence, and that the new dsRNA sequence had a low off-target probability score calculated by dsCheck [22]. Using the second lines, lectin analysis was independently repeated (*n* = 2), and the values between the knockdown and control (*GMR>GFP^RNAi^*) were tested by two-sided Student's *t*-tests. Genes with *p*<0.05 were considered glycosylation-related genes (Rank 1). When no change in lectin values was observed solely using *GMR-Gal4*, the examination was repeated using *GMR-Gal4; UAS-dicer2* (VDRC). RNAi flies from VDRC whose target sequence had no overlap with the primary construct were also used as second lines.

For candidate genes that were not evaluated with the second lines, first the number of 19mers with a perfect match to each *Drosophila* transcript was calculated using dsCheck. The highest number of 19mers with a perfect match to the off-target gene was defined as the “off-target probability score”. The genes with a score below 3 were tested to determine whether knockdown of their suspected off-target genes led to glycosylation defects. When the knockdown of the suspected off-target genes did not lead to any evidence of glycosylation defects, the corresponding genes were added to the list of genes required for glycosylation (Rank 2).

### GO analysis

Our final glycosylation hit list was annotated to include information on protein domain, molecular function, and protein family by cross-referencing InterPro (version 20.0 and 21.0) [47], Flybase, and Panther [48]. On the basis of the resulting annotation, genes were manually classified into 9 categories. These annotations are shown in Table S4.

### Mass spectrometry (MS) analysis of glycopeptides and glycan structure

MS analysis of glycopeptides was performed as previously described [16]. Purified Chp was digested with modified trypsin (Promega). Glycopeptides and peptide mixtures were separated by reversed-phase high-performance liquid chromatography (RP-HPLC) using an Inertsil C18 column (GL Science). The matrices for peptide analysis by matrix-assisted laser desorption/ionization time-of-flight MS (MALDI-TOF-MS) spectra were acquired using a Voyager mass spectrometer (Applied Biosystems). MS/MS measurements were performed using an Ultraflex TOF/TOF mass spectrometer with the LIFT-MS/MS facility (Bruker Daltonics GmbH).

### Immunostaining

To investigate the amount of α1,3-fucosylation *in vivo*, frozen sections of adult eye demonstrating *swm* or *gfp* knockdown were prepared. Immunostaining was performed as previously described [49]. Fluorescent images were acquired with a laser scanning confocal microscope, LSM700 (Zeiss).

### Cell culture, plasmid construction, transfection, and siRNA treatment

BG2-c6 cells were cultured in M3 medium (Sigma) containing 10% FBS and 10 µg/mL insulin (Invitrogen) at 25°C. Transfection was performed using a calcium phosphate transfection kit (Invitrogen).

To construct an expression plasmid for C-terminal Flag-tagged Swm (Swm-Flag), cDNA containing the *swm* coding region was obtained from *Canton-S* total RNA by RT-PCR. A DNA fragment encoding the *3Flag* sequence was ligated to the 3′end of the *swm* coding region and inserted into the expression vector pRmHa.

For the transient expression of Swm-Flag, 1.5 µg plasmid was added to each well of a 24-well plate, in which 5×10^5^ BG2-c6 cells had been seeded. Plasmid expression was induced by incubating cells in the presence of 0.1–0.5 mM CuSO_4_ for periods from 3 h to overnight.

For knockdown experiments, dsRNA for *swm* or *gfp* was produced using *in vitro* transcription T7 kit (Takara). A 3-µg aliquot of dsRNA was added to each well of a 24-well plate in which 5×10^5^ cells had been seeded. After 5 days, cells were harvested for the following assays.

### Immunoblot

For protein analysis, equal amounts of protein extracted from fly eyes were subjected to SDS-PAGE. After transfer to PVDF membranes (Millipore), membranes were blocked in PBS containing 0.05% Tween 20 and 5% skim milk and incubated overnight with the primary antibody, followed by the secondary antibody. A monoclonal anti-α-tubulin antibody was used for normalization. Detection was performed using the Supersignal West Pico Chemiluminescent Substrate.

### RNP-IP assay

Immunoprecipitation of protein–RNA complexes was performed according to a protocol by Niranjanakumari et al. [50]. BG2-c6 cells transfected with *swm-Flag* were harvested and cross-linked in PBS containing 0.2% PFA. Following sonication in RIPA buffer (50 mM Tris-HCl pH 7.5, 1% NP-40, 0.05% SDS, 1 mM EDTA, 150 mM NaCl) with protease inhibitor (Nakarai), each supernatant was mixed with a monoclonal anti-Flag M2 antibody or normal mouse IgG and then incubated with protein-G beads (GE Healthcare) at 4°C for 6 h. Sepharose beads were washed 5 times with RIPA buffer and incubated in 50 mM Tris (pH 7.0), 5 mM EDTA, 10 mM DTT, and 1% SDS at 70°C for 45 min to reverse cross-linking. RNA was extracted from the immunoprecipitates using Sepasol reagent (Nakarai) followed by DNase I treatment. The extracted RNA was used as a template to synthesize cDNA using Superscript III (Invitrogen) according to the manufacturer's protocol.

### Real-time PCR

Total RNA was extracted using the RNeasy mini kit (Qiagen) or Sepasol reagent (Nakarai). Complementary DNAs were synthesized using Superscript III according to the manufacturer's protocol. Real-time PCR was carried out using a 7900HT Fast Real Time PCR system (Applied Biosystems) with Power Syber Green. Samples were normalized with Drosophila *rpl32*.

### RNA degradation analysis

BG2-c6 cells transfected with *gfp* or *swm* dsRNA were grown for 5 days, and then actinomycin D (Nakarai) was added to a final concentration of 5 µg/mL to arrest *de novo* RNA synthesis. At 30, 60, and 90 min after actinomycin D treatment, the cells were harvested, and *fucTA* and *rpl32* (control) mRNA was quantified by real-time PCR as described above.

### PAT assay

PolyA measurement of *fucTA* mRNA was carried out as described [51]. PCR fragments from the PAT assay were separated on 2% agarose gels and visualized by Southern blot using DIG DNA Labeling and Detection kit (Roche).

### Subcellular fractionation

Fractionation and RNA isolation were performed as described [52]. The cytoplasmic fraction was extracted by hypotonic buffer (10 mM Tris (pH 7.5), 10 mM NaCl, 3 mM MgCl_2_, 0.5% (v/v) Nonident P-40, 40 units/mL RNase inhibitor, 1 mM DTT) and then extracted by EDTA buffer (10 mM Tris (pH 7.5), 10 mM NaCl, 40 mM EDTA, 0.5% (v/v) Nonident P-40, 40 units/mL RNase inhibitor, 1 mM DTT). The remaining pellet was used as the nuclear fraction. Sepasol solution was used to purify mRNA from each fraction as described above.

### Human homologs

The InParanoid database (version 7.0) was searched for mammalian homologs [53].

## Supporting Information

Figure S1 Schemes of Chp glycan attachment sites and glycan structures. (upper) The glycosylated sites in Chp are indicated as ‘Y’ with amino acid positions. The colors represent the glycan types. Open boxes in Chp protein indicate leucine-rich repeats. (lower) Schemes for three types of glycan structures are represented. The number of sugars is indicated in parentheses following the sugar name. The moieties recognized by lectins are encircled by lines colored to indicate the lectin species.
(9.83 MB TIF)

Figure S2 An example of dot blot analysis for the screen. Chp purified from adult heads with the knockdown of each gene (1A∼11H) and the control *gfp* (12A∼12H) was blotted and detected with anti-Chp (left) and anti-HRP (right). The control Chp was sequentially diluted and blotted at the points from 12A to 12H. Dots in red squares represent anti-Chp and anti-HRP signals against Chp purified from *swm* knockdown flies.
(4.28 MB TIF)

Figure S3 Glycan structures attached to N1012 in Chp. Six glycan structures that were identified by mass spectrometry analysis are schematically represented.
(5.23 MB TIF)

Figure S4 Knock-in of the *fucTA* gene by ends-out recombination. (A) The donor DNA was generated by FLP and I-SceI action from the X chromosome (top). Homologous recombination was used to insert the 3Myc sequence into the endogenous *fucTA* gene and the *white* gene into the region between *fucTA* and *Msh6* genes (middle). The expected structure (bottom) was verified by PCR with two sets of primers (colored thick arrows). (B) Expected bands were amplified with two sets of primers (red, *fucTA* locus; blue, *Msh6* locus) using chromosomal DNA from a *fucTA* knock-in fly (*fucTA-Myc^KI^*) but not in *white* flies (*w*). (C) Myc-tagged FucTA protein (arrow) was detected by immunoblot using anti-Myc antibody in the adult eye extracts of *fucTA* knock-in flies (*fucTA-Myc^KI^*) but not in *white* flies (*w*). The amount of Myc-tagged FucTA protein was reduced in *swm* knockdown eyes (*swm* KD) compared to the control eyes (*gfp* KD).
(8.80 MB TIF)

Figure S5 The amount of *Mgat1* and *fdl* mRNA in *swm* knockdown BG2-c6 cells. The amount of mRNA encoding *Mgat1* was slightly but significantly increased, whereas that of *fdl* mRNA was not changed in *swm* knockdown cells compared with control *gfp* knockdown cells, *n*  =  3. *p < 0.05.
(1.77 MB TIF)

Table S1 Specificity of the lectins used in this study.
(0.02 MB XLS)

Table S2 Glycosylation Screening Results using NIG RNAi fly strains.
(1.39 MB XLS)

Table S3 Glycosylation Screening Results using VDRC RNAi fly strains.
(0.06 MB XLS)

Table S4 Glycosylation-related genes identified in this study.
(0.08 MB XLS)

Table S5 Manmalian homolog.
(0.03 MB XLS)

Table S6 Primer Sequences for transgenic fly.
(0.03 MB XLS)

Table S7 Primer Sequences used in this study in cultured cells.
(0.02 MB XLS)
