# Supplementary figures and images for: Identification of Genes Required for Neural-Specific Glycosylation Using Functional Genomics
Source: PLoS Genet. 2010 Dec 23;6(12):e1001254. doi: 10.1371/journal.pgen.1001254 (PMC3009669; doi:10.1371/journal.pgen.1001254)

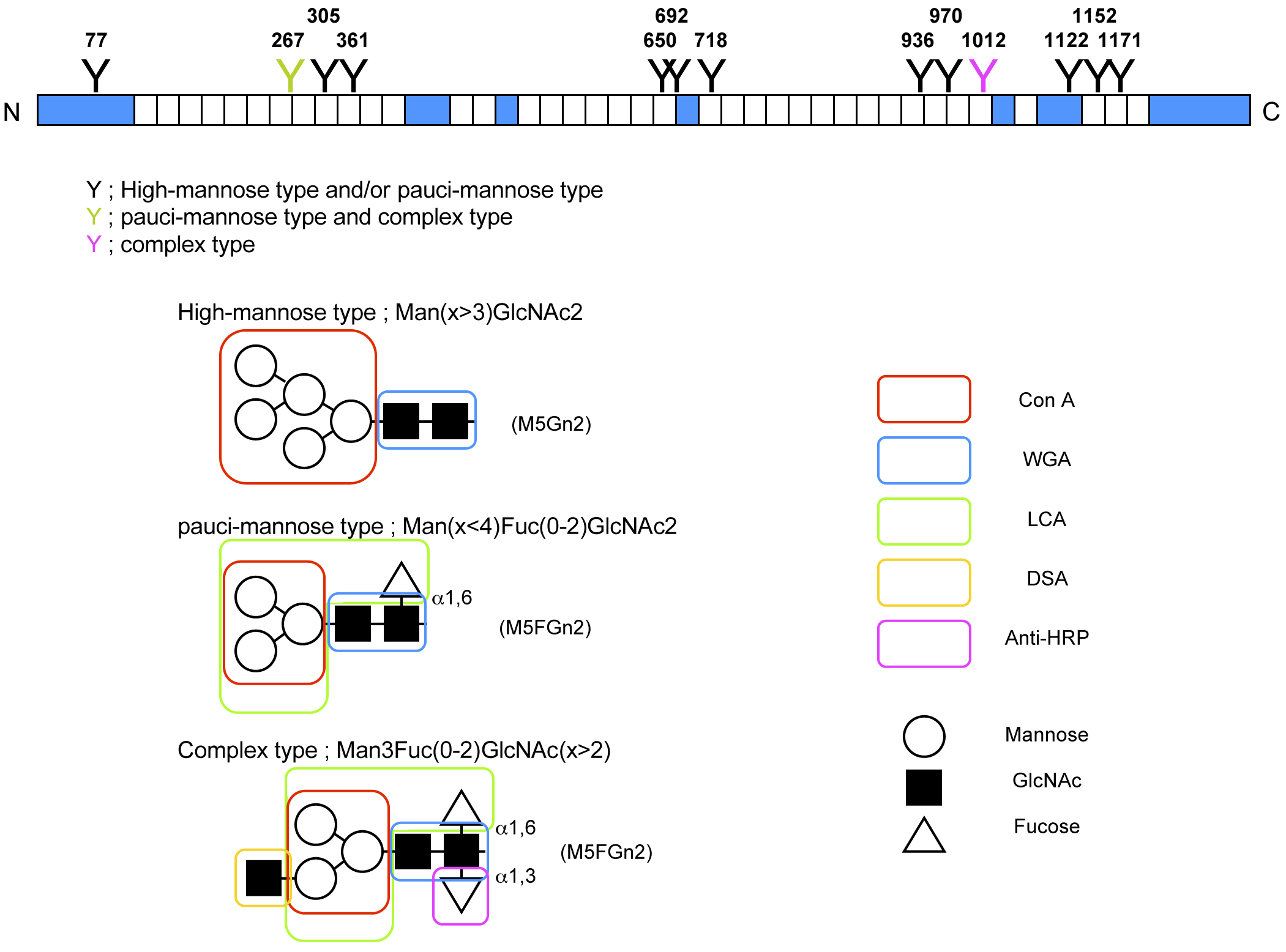

Supplement: Figure S1 — Schemes of Chp glycan attachment sites and glycan structures. (upper) The glycosylated sites in Chp are indicated as ‘Y’ with amino acid positions. The colors represent the glycan types. Open boxes in Chp protein indicate leucine-rich repeats. (lower) Schemes for three types of glycan structures are represented. The number of sugars is indicated in parentheses following the sugar name. The moieties recognized by lectins are encircled by lines colored to indicate the lectin species. (9.83 MB TIF) [file pgen.1001254.s001.tif]

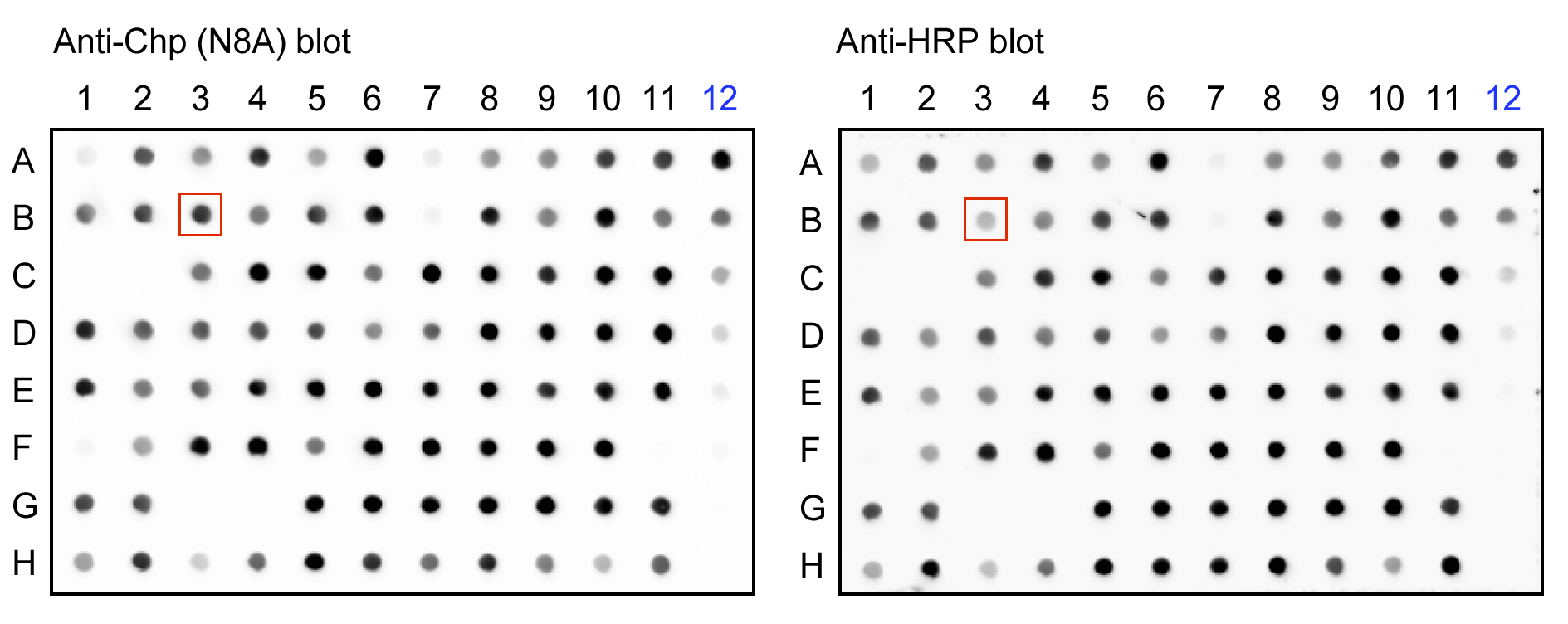

Supplement: Figure S2 — An example of dot blot analysis for the screen. Chp purified from adult heads with the knockdown of each gene (1A∼11H) and the control gfp (12A∼12H) was blotted and detected with anti-Chp (left) and anti-HRP (right). The control Chp was sequentially diluted and blotted at the points from 12A to 12H. Dots in red squares represent anti-Chp and anti-HRP signals against Chp purified from swm knockdown flies. (4.28 MB TIF) [file pgen.1001254.s002.tif]

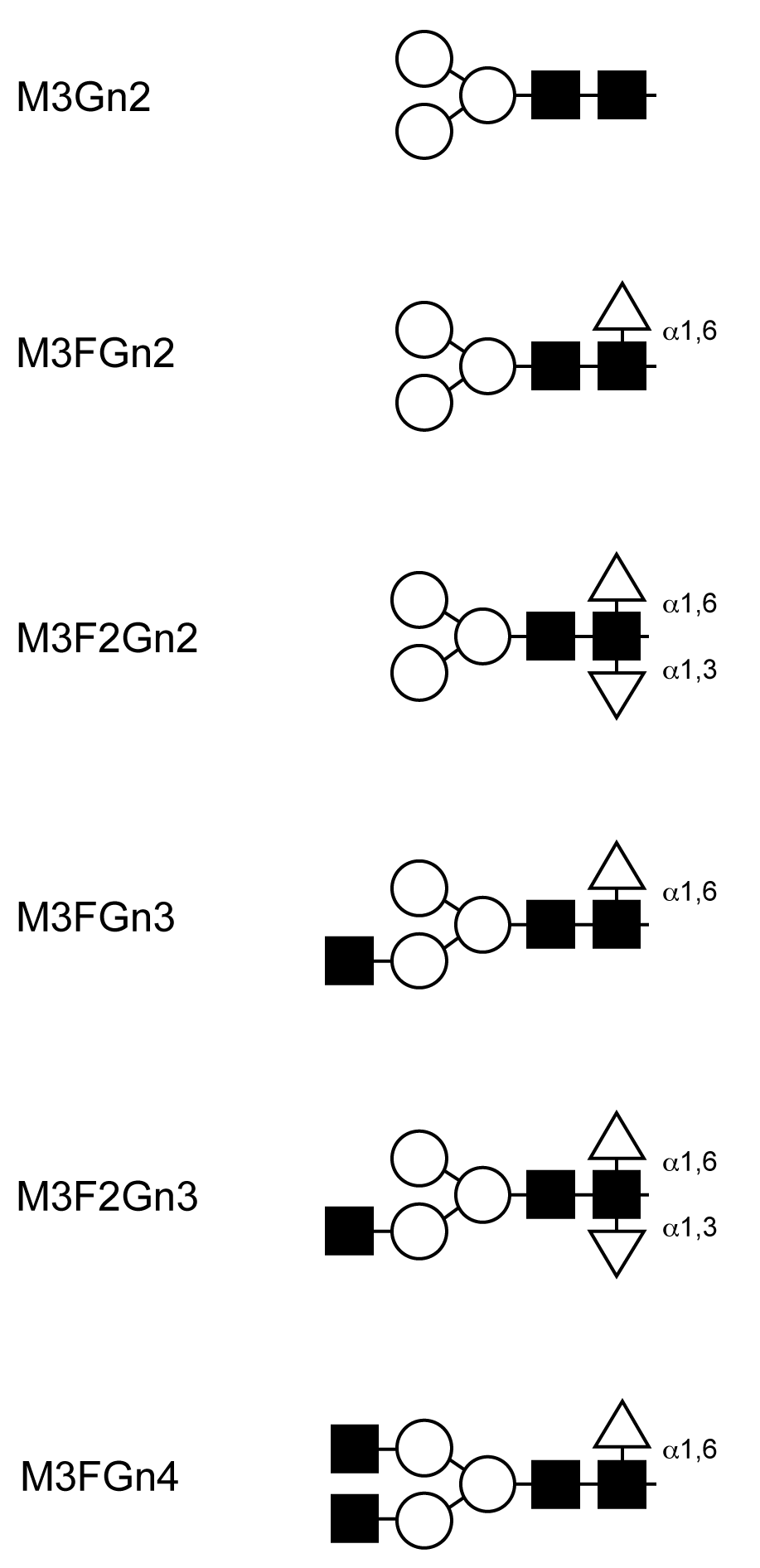

Supplement: Figure S3 — Glycan structures attached to N1012 in Chp. Six glycan structures that were identified by mass spectrometry analysis are schematically represented. (5.23 MB TIF) [file pgen.1001254.s003.tif]

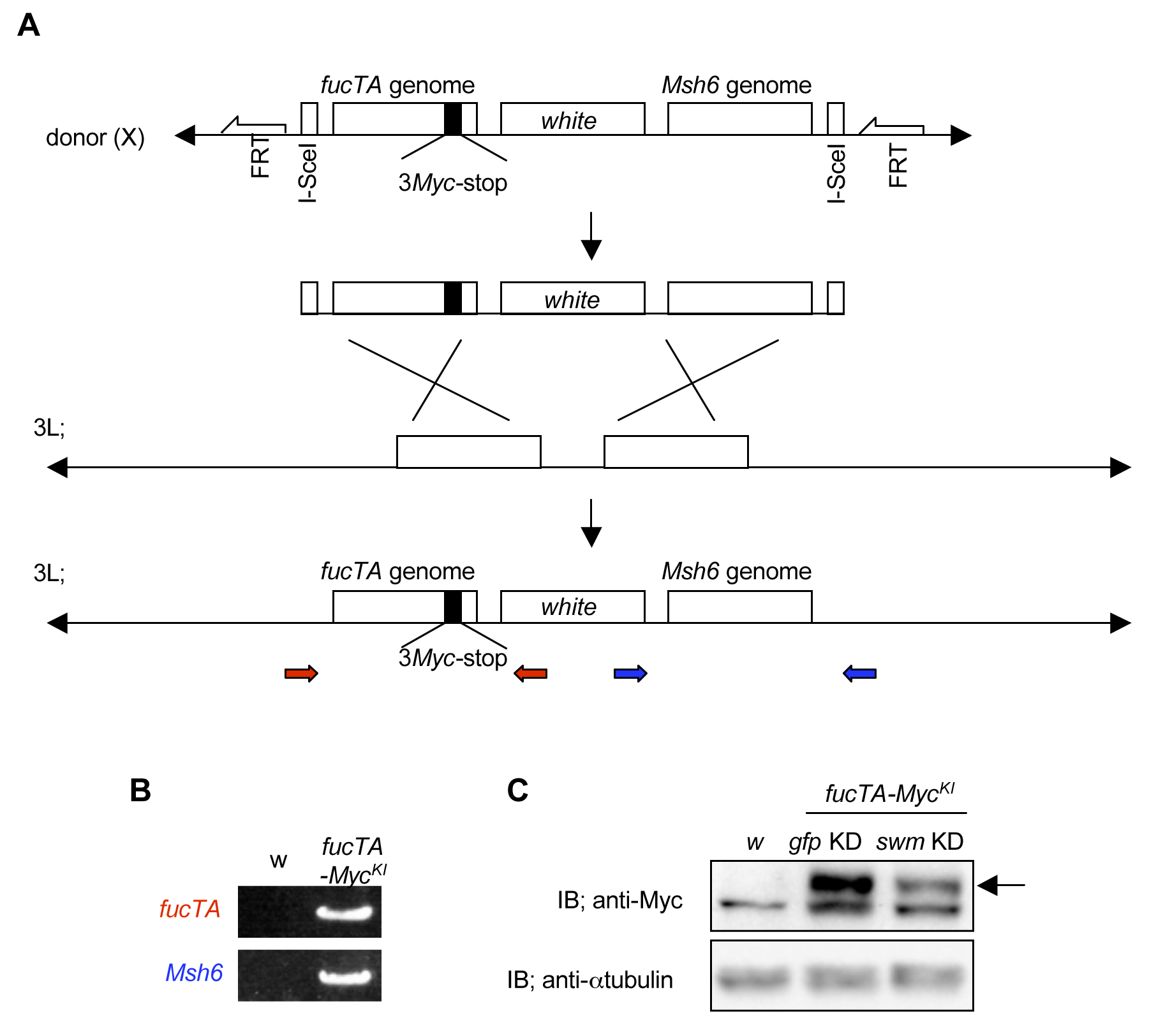

Supplement: Figure S4 — Knock-in of the fucTA gene by ends-out recombination. (A) The donor DNA was generated by FLP and I-SceI action from the X chromosome (top). Homologous recombination was used to insert the 3Myc sequence into the endogenous fucTA gene and the white gene into the region between fucTA and Msh6 genes (middle). The expected structure (bottom) was verified by PCR with two sets of primers (colored thick arrows). (B) Expected bands were amplified with two sets of primers (red, fucTA locus; blue, Msh6 locus) using chromosomal DNA from a fucTA knock-in fly (fucTA-MycKI) but not in white flies (w). (C) Myc-tagged FucTA protein (arrow) was detected by immunoblot using anti-Myc antibody in the adult eye extracts of fucTA knock-in flies (fucTA-MycKI) but not in white flies (w). The amount of Myc-tagged FucTA protein was reduced in swm knockdown eyes (swm KD) compared to the control eyes (gfp KD). (8.80 MB TIF) [file pgen.1001254.s004.tif]

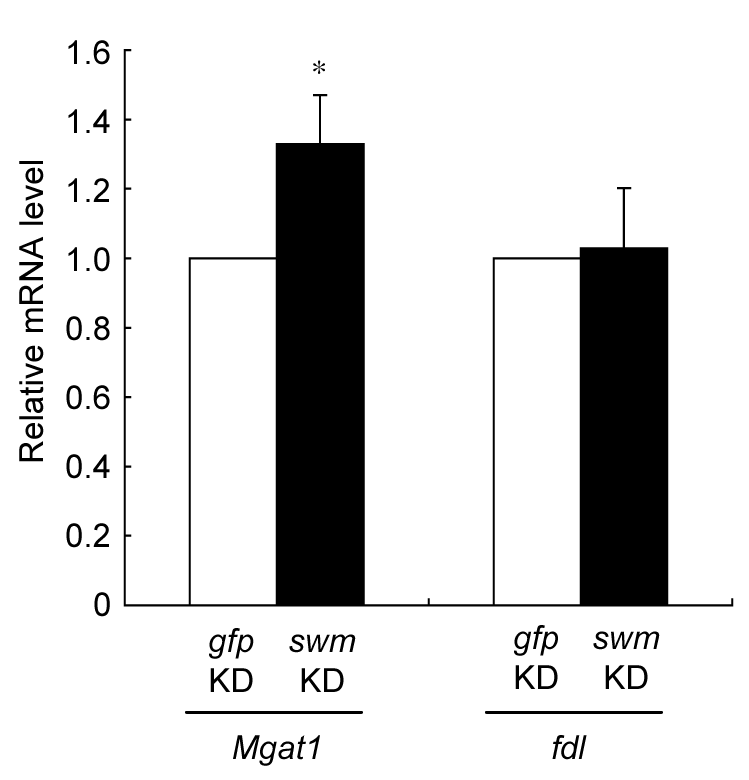

Supplement: Figure S5 — The amount of Mgat1 and fdl mRNA in swm knockdown BG2-c6 cells. The amount of mRNA encoding Mgat1 was slightly but significantly increased, whereas that of fdl mRNA was not changed in swm knockdown cells compared with control gfp knockdown cells, n = 3. *p < 0.05. (1.77 MB TIF) [file pgen.1001254.s005.tif]
